# Robotic-Assisted Colovesical Fistula Repair: A Case Report

**DOI:** 10.7759/cureus.54404

**Published:** 2024-02-18

**Authors:** Shailesh C Sahay, Vivek Mangla, Pawan Kesarwani, Abhigyan Goel

**Affiliations:** 1 Urology, Max Super Speciality Hospital, Patparganj, New Delhi, IND; 2 Gastrointestinal Surgery, Max Super Speciality Hospital, Patparganj, New Delhi, IND

**Keywords:** robotic-assisted surgery, enterovesical fistula, urology surgery, robotic repair, sigmoidectomy, case report, diverticular disease, colovesical fistula

## Abstract

Colovesical fistulas present a diagnostic and therapeutic challenge, commonly arising from complications of diverticular disease. In our case, a 71-year-old male with colovesical fistula symptoms underwent robotic-assisted surgery for complicated sigmoid diverticulitis. Intraoperatively, meticulous adhesiolysis and fistula repair were performed. Histopathology confirmed diverticular disease. Postoperatively, the patient recovered well. Colovesical fistulas may indicate underlying malignancy in diverticulitis. With a lack of standardized protocols, our case suggests that robotic-assisted surgery offers improved outcomes, better vision, and ergonomics. To conclude, robotic-assisted colovesical fistula repair and sigmoidectomy demonstrated excellent outcomes, suggesting a promising approach for enhanced postoperative recovery.

## Introduction

Enterovesical fistulae, though rare, are on the rise [1]. Colovesical fistulas represent the majority, at 95% [2]. Colovesical fistula, an abnormal connection between the colon and urinary bladder, poses a multifaceted challenge in both diagnosis and management. This unique pathological entity often arises from complications of inflammatory, neoplastic, or diverticular diseases. Diverticular disease is the most common [3]. Most cases present with frequent UTIs and pneumaturia (the most sensitive and specific), sometimes with fecaluria as well [3]. It is difficult to treat them conservatively. Some small fistulae may respond to indwelling Foley's catheter placement or urinary diversion by percutaneous nephrostomy. After surgical repair of the fistula, patients reported a high satisfaction score of 3.6 out of 4, as it led to the resolution of symptoms in most cases, regardless of postoperative complications [4].

## Case presentation

**Figure 1:**
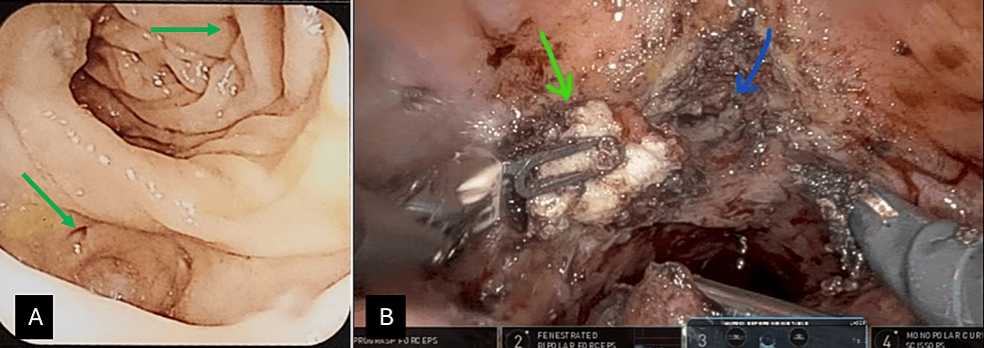
(A) Colonoscopy image showing sigmoid colitis with a mouth of the diverticula (green arrows). (B) Laparoscopic view of the abdomen showing colovesical fistula (green arrow: colovesical fistula; blue arrow: mouth of the fistula)

The index patient was a 71-year-old male who presented with symptoms of the passage of air and fecal material in urine for six months. He also had a history of recurrent urinary and intestinal infections, leading to altered bowel habits. He was examined by a gastroenterologist, who performed a colonoscopy. A colonoscopy revealed sigmoid diverticulitis with multiple diverticula in the sigmoid and descending colons, along with hemorrhoids. There was no obvious tumor/growth in the colon. There was no fistulous opening found at colonoscopy (Figure 1A).

**Figure 2:**
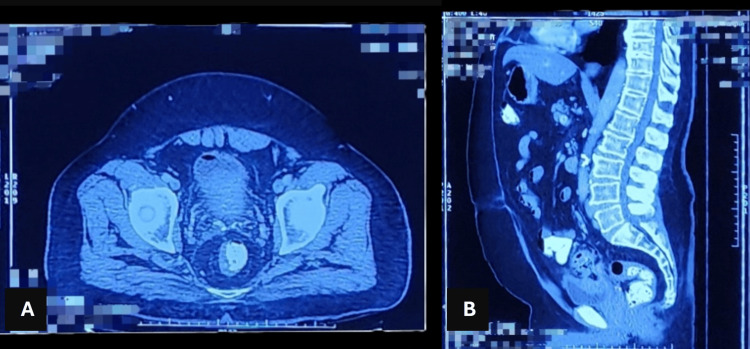
(A) CT transverse image showing air pocket in the bladder. (B) CT coronal image CT: computed tomography

Contrast-enhanced CT of the whole abdomen revealed extensive, circumferential, mildly enhancing hypodense wall thickening in the sigmoid colon, with a maximum thickness of 17 mm (Figure 2A-2B). Multiple small, air-filled diverticula arising from the sigmoid colon, accompanied by pronounced mesocolonic fat stranding and congestion of the vasa recta, were seen. A heterogeneous area measuring 38 x 35 mm, exhibiting peripheral enhancing walls and internal air pockets, was noted near the bladder dome, suggesting a potential fistulous communication with the adjacent sigmoid colon with thickening of the urinary bladder walls and the presence of air pockets in the urinary bladder. Additionally, mild wall thickening was identified in the proximal rectum and descending colon. These findings were indicative of complicated sigmoid diverticulitis with a likely colovesical fistula.

The pre-operative assessment via cystoscopy revealed a normal urethra, a Grade 2 prostate, normal bilateral ureteric orifices, and diffuse cystitis. Additionally, fecal particles were observed within the bladder lumen, and cystitis extended up to the trigone, necessitating the placement of bilateral double-J (DJ) stents. The ports were placed for pelvic surgery by the da Vinci Xi robotic system (Intuitive Surgical, Inc., Sunnyvale, California). All four robotic ports of 8 mm size were placed in a horizontal line at the level of the umbilicus, 8 cm apart. The robotic approach had the advantage of 3D vision and better reachability in the deeper pelvis due to the seven degrees of freedom of its instruments. Intraoperatively, dense adhesions were identified between the sigmoid colon and the bladder dome and posterior bladder wall. Skillful adhesiolysis and dissection were performed to separate the structures. The colovesical fistula was dismantled, and surrounding tissue at the urinary bladder fistula site was excised to freshen the area for suturing. The excised specimen was sent for biopsy (Figure 1B). The repair of the bladder was executed in two layers, utilizing 3-0 V-Loc (Medtronic, Minnesota, USA) sutures. A subsequent bladder inflation test was done with 200 ml of saline solution with methylene blue in a 10:1 ratio through the Foley catheter to rule out any leakage from the sutured wall of the bladder near the dome. Following the successful completion of the procedure, the case was transitioned to the gastrointestinal surgery team for further management. The sigmoid colon was removed from the descending colon until the rectosigmoid junction. The sigmoid colectomy was done with the help of a gelport, and the colorectal anastomosis was done intracorporeally with Endo GIA and EEA staplers (Medtronic, Minnesota, USA). Diverting loop ileostomy was done as there were dense adhesions around the rectum and the rectal mucosa was inflamed. A single pelvic drain was placed for 48 hours. Total blood loss during surgery was approximately 250 ml, and the total time taken was 4.5 hours. The histopathological examination was consistent with a complicated diverticular disease. Postoperative recovery was uneventful without any complications. He was discharged on the fourth post-operative day on an oral-normal diet. The patient was residing in a distant place from the hospital, so he came for stent removal and ileostomy closure after one month. His distal loopogram through the distal loop of the ileostomy was done along with a contrast enema study at one month, which showed no leakage of contrast from the anastomotic site. The distal loopogram is not a routine investigation in all the ileostomy closure procedures, but it was done because the rectum was inflamed and there was a fear of anastomotic leakage. His Foley catheter was removed at three weeks. Ileostomy closure, cystoscopy, and DJ stent removal were done in one month. His bladder was found to be healthy on cystoscopy, and the fistula was in the healing phase. His symptoms totally resolved, and he did not have any urinary infections until one year of follow-up.

## Discussion

In our case, the patient's presentation of a colovesical fistula was associated with sigmoid diverticulitis, a condition with potential concomitant malignancy. Studies suggest a 3% to 5% incidence of malignancy in uncomplicated diverticulitis and an 11% incidence for complicated cases [5]. Considering the high recurrence rate of up to 40% in complicated diverticulitis, elective colon resection becomes a crucial intervention [3]. This patient had a history of passing fecal material in urine, leading to recurrent urinary tract infections as well as colitis; hence, a sigmoid colectomy was performed here. Notably, conservative management is associated with low closure rates, prompting consideration for surgical interventions, although there are no standardized protocols for such repairs [6]. Using dedicated robotic platforms in surgery enhances precision and flexibility, thus making it easy to dissect deeper pelvic areas. However, the surgical outcomes of open, laparoscopic, and robotic approaches are comparable [7]. Robotics provides superior visualization, dexterity, and ergonomics compared to traditional endoscopic surgery while still maintaining the advantages of minimally invasive techniques [8]. Our contribution through this case report aims to add valuable insights about the possibility of a robotic approach to managing such fistulas. However, there is no evidence in the literature to compare the different approaches and their outcomes in colovesical fistula management. Robotic-assisted colectomy and colovesical fistula management may enhance outcomes for individuals with colovesical fistulas. We need to have more cases to compare the efficacy and safety of the robotic approach to managing such complex cases.

## Conclusions

Colovesical fistulas, though rare, are witnessing an increase in incidence. Diverticular disease remains the most common etiology. Symptoms often manifest as recurrent urinary infections, pneumaturia, and occasionally fecaluria. Conservative management proves difficult, whereas surgical repair yields high patient satisfaction as it resolves symptoms in most cases. The present case was associated with sigmoid diverticulitis with dense adhesions around the colon and rectum. Robotic-assisted surgical repair helped in reaching the deeper pelvic areas easily due to its 3D vision, and the greater ergonomics of the instruments added to its advantage. However, we need more evidence to support the potential of this novel approach for optimizing patient care.
